# PCP4/PEP19 promotes migration, invasion and adhesion in human breast cancer MCF-7 and T47D cells

**DOI:** 10.18632/oncotarget.7529

**Published:** 2016-02-20

**Authors:** Takuya Yoshimura, Taiji Hamada, Hiroshi Hijioka, Masakazu Souda, Kazuhito Hatanaka, Takako Yoshioka, Sohsuke Yamada, Masato Tsutsui, Yoshihisa Umekita, Norifumi Nakamura, Akihide Tanimoto

**Affiliations:** ^1^ Department of Oral and Maxillofacial Surgery, Kagoshima University Graduate School of Medical and Dental Sciences, Kagoshima, Japan; ^2^ Department of Pathology, Kagoshima University Graduate School of Medical and Dental Sciences, Kagoshima, Japan; ^3^ Department of Pathology, Children's Cancer Center, National Center for Child Health and Development, Tokyo, Japan; ^4^ Department of Pharmacology, Faculty of Medicine, University of the Ryukyus, Okinawa, Japan; ^5^ Division of Organ Pathology, Faculty of Medicine, Tottori University, Yonago, Japan

**Keywords:** PCP4/PEP19, breast cancer, EMT, migration, invasion

## Abstract

Purkinje cell protein (PCP) 4/peptide (PEP) 19 is expressed in Purkinje cells where it has a calmodulin-binding, anti-apoptotic function. We recently demonstrated that PCP4/PEP19 is expressed and inhibit apoptosis in human breast cancer cell lines. In the present study we investigated the role of PCP4/PEP19 in cell morphology, adhesion, migration, and invasion in MCF-7 and T47D human breast cancer cell lines. Knockdown of PCP4/PEP19 reduced the formation of filopodia-like cytoplasmic structures and vinculin expression, and enhanced E-cadherin expression. Activities of migration, invasion, and cell adhesion were also decreased after the knockdown of PCP4/PEP19 in MCF-7 and T47D cells. These results suggested that PCP4/PEP19 promotes cancer cell adhesion, migration, and invasion and that PCP4/PEP19 may be a potential target for therapeutic agents in breast cancer treatment which act by inhibiting epithelial-mesenchymal transition and enhancing apoptotic cell death.

## INTRODUCTION

Purkinje cell protein (PCP) 4, also known as peptide (PEP) 19, was first identified in rat cerebellum as a polypeptide of 7.6 kDa that shows homology to the calcium binding β-chain of the S100 protein [1]. It is expressed in Purkinje cells and stellate neurons [2] and binds calmodulin (CaM), thereby regulating CaM-dependent signaling [3–5] by modulating calcium/CaM-dependent protein kinase (CaMK) activity [6, 7] to influence a variety of processes in neurons, including apoptosis [8–11].

Although PCP4/PEP19 was first detected in the central nervous system, it is also known to be expressed in other organs, including prostate, kidney, and uterus as well as breast cancer tissue. PCP4/PEP19 are highly expressed in uterine leiomyoma as compared to non-neoplastic normal myometrium [12]. Another study reported PCP4/PEP19 expression in normal and neoplastic human adrenocortical tissue where it increases aldosterone production [13, 14]. Our previous study showed that PCP4/PEP19 expression was increased in the mammary gland tissue of rats in which carcinogenesis was induced by exposure to 7, 12-dimethylbenz [a] anthracene exposure [15]. We also detected PCP4/PEP19 expression in human breast cancer and found that it exerts anti-apoptotic functions in human breast cancer cell lines via CaMKK and Akt signaling pathways [16].

B cell-specific Moloney murine leukemia virus integration site (Bmi)-1 is a critical inducer of cell adhesion, migration, and invasion as well as the epithelial-mesenchymal transition (EMT) in human breast cancer cells [17–19]. In the present study, we investigated the roles of PCP4/PEP19 and Bmi-1 in cell adhesion, migration, and invasion by knocking down their transcripts and evaluating the expression of cell adhesion complex components as well as migratory and invasive capacities in human breast cancer MCF-7 and T47D cells. The activities of Ras homolog (Rho) A, Ras-related C3 botulinum toxin substrate (Rac) 1, and cell division cycle (Cdc) 42 GTPases were also assayed in MCF-7 cells. We found that these cellular processes were inhibited by PCP4/PEP19 knockdown through a mechanism(s) distinct from Bmi-1 signaling, indicating that PCP4/PEP19 enhances cell motility and prevent apoptosis, and therefore can determine malignant potential of cancer cells.

## RESULTS

### PCP4/PEP19 induction by 17β-estradiol (E2) and subcellular localization

**Figure 1:**
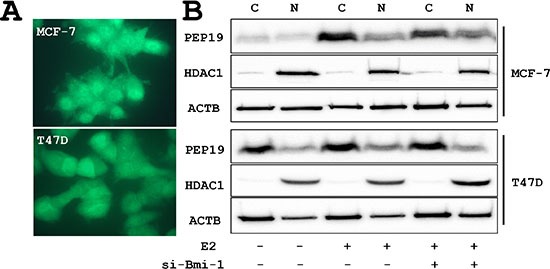
PCP4/PEP19 expression and subcellular localization (**A**) Immunocytochemical demonstration of cytoplasmic and nuclear localization of PCP4/PEP19 in MCF-7 and T47D cells. (**B**) Subcellular fractionation of PCP4/PEP19 expression by Western blotting. PCP4/PEP19 localized in both cytoplasmic (**C**) and nuclear (N) fraction before and after Bmi-1 knockdown. The PCP4/PEP19 expression was induced by E2 stimulation (10^−9^ M E2 for 48 hr after 24 hr E2-free starvation). si-Bmi-1, Bmi-1 knockdown; N, nuclear fraction; C, cytoplasmic fraction; ACTB, β-actin; HDAC1, histone deacetylase 1.

Immunocytochemistry showed that PCP4/PEP19 was localized in both cytoplasm and nuclei in MCF-7 and T47D cells (Figure 1A). The expression of PCP4/PEP19 was induced by E2 stimulation in both cells. The cytoplasmic and nuclear localization was confirmed by Western blotting and the subcellular localization was not changed after E2 stimulation and Bmi-1 knockdown (Figure 1B).

### Bmi-1 and PCP4/PEP19 knockdown alters cell morphology

**Figure 2:**
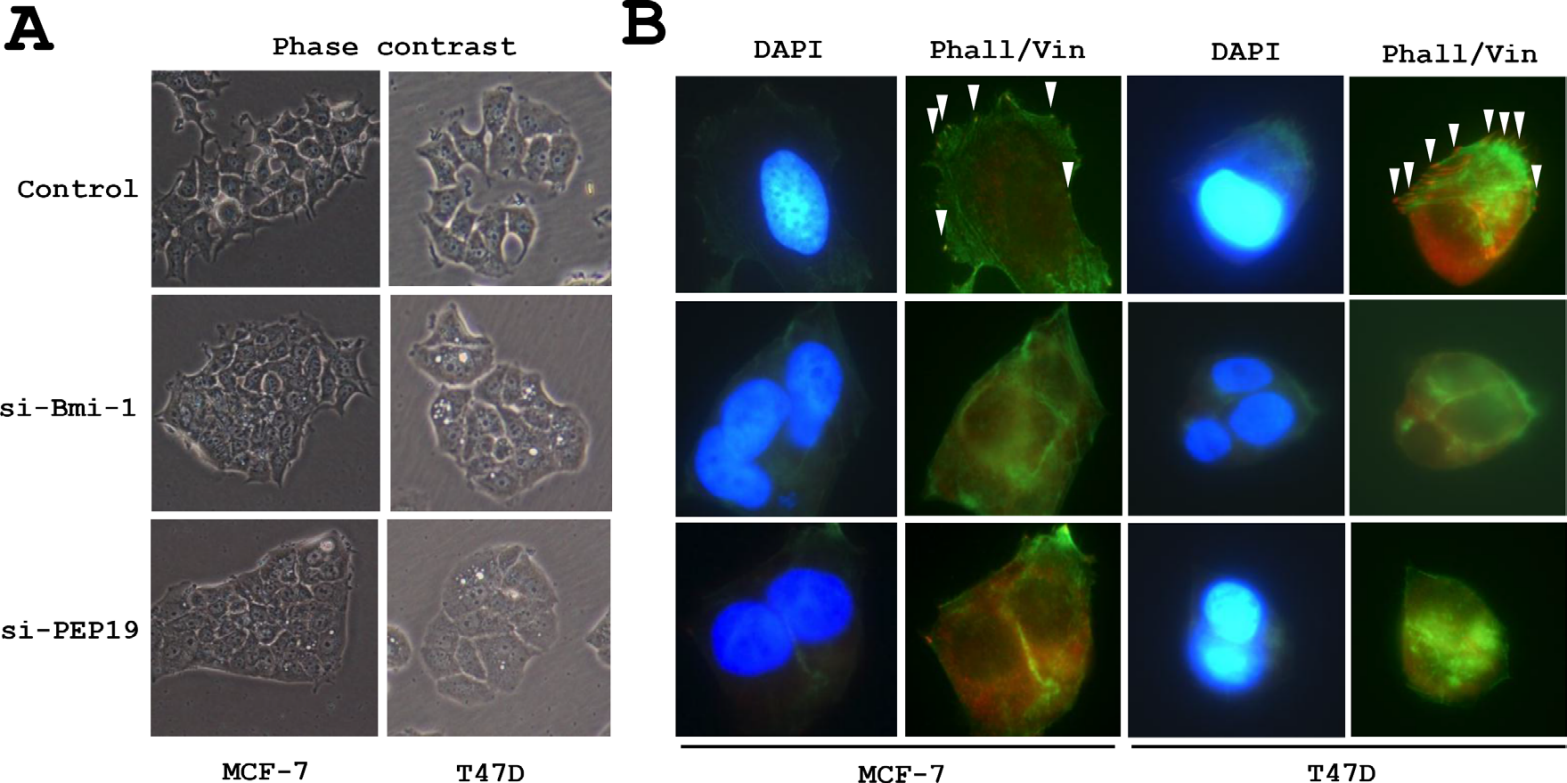
Changes in cell morphology following PCP4/PEP19 and Bmi-1 knockdown (**A**) Phase contrast images of MCF-7 and T47D cells. Bmi-1 and PCP4/PEP19 knockdown cells were aggregated and had fewer filopodia-like structures. (**B**) F-actin organization and vinculin expression (arrowheads) as detected by FITC-phalloidin staining and immunocytochemistry, respectively, were lost after Bmi-1 and PCP4/PEP19 knockdown. The cells were stimulated with 10^−8^ M E2 for 15 min after overnight starvation. si-Bmi-1, Bmi-1 knockdown; si-PEP19, PCP4/PEP19 knockdown; DAPI, 4′, 6-diamidino-2-phenylindole: Phall, phalloidin; Vin, vinculin.

**Figure 3:**
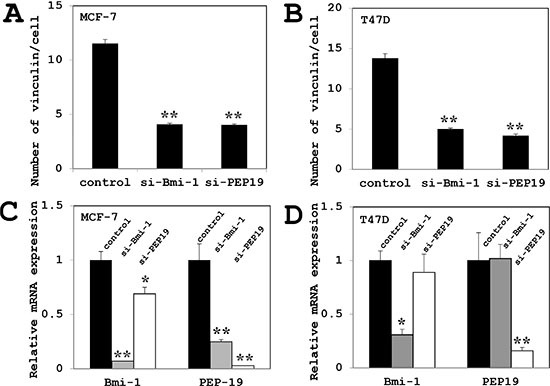
Effects of Bmi-1 and PCP4/PEP19 knockdown on vinculin expression and PCP4/PEP19 and Bmi-1 mRNA expression (**A**–**B**) Quantitative analysis of vinculin-positive structures in each cell (*n* = 100) of MCF-7 cells (A) and T47D cells (B). The number was decreased after Bmi-1 and PCP4/PEP19 knockdown as compared to controls. (**C**–**D**) PCP4/PEP19 expression was decreased by Bmi-1 knockdown, while Bmi-1 expression was reduced by PCP4/PEP19 knockdown (*n* = 4) in MCF-7 cells (A). In contrast, PCP4/PEP19 and Bmi-1 expression were not influenced each other in T47D cells (B). *p < 0.05 and **p < 0.01 vs. control. si-Bmi-1, Bmi-1 knockdown; si-PEP19, PCP4/PEP19 knockdown.

Cell morphology was altered by siRNA-mediated Bmi-1 and PCP4/PEP19 knockdown; MCF-7 and T47D cells appeared rounded, had fewer cytoplasmic processes, and were more densely packed into aggregates. The number of filopodia-like cytoplasmic projections per cell were decreased in Bmi-1 and PCP4/PEP19 knockdown cells (Figure 2A), and imuunocytochemical analysis of vinculin expression showed rod- or spot-like structures within cytoplasmic processes (Figure 2B) corresponding to focal adhesion complexes, which were also decreased in number (Figure 3A and 3B).

### Expression of EMT markers is altered upon Bmi-1 and PCP4/PEP19 knockdown

**Figure 4:**
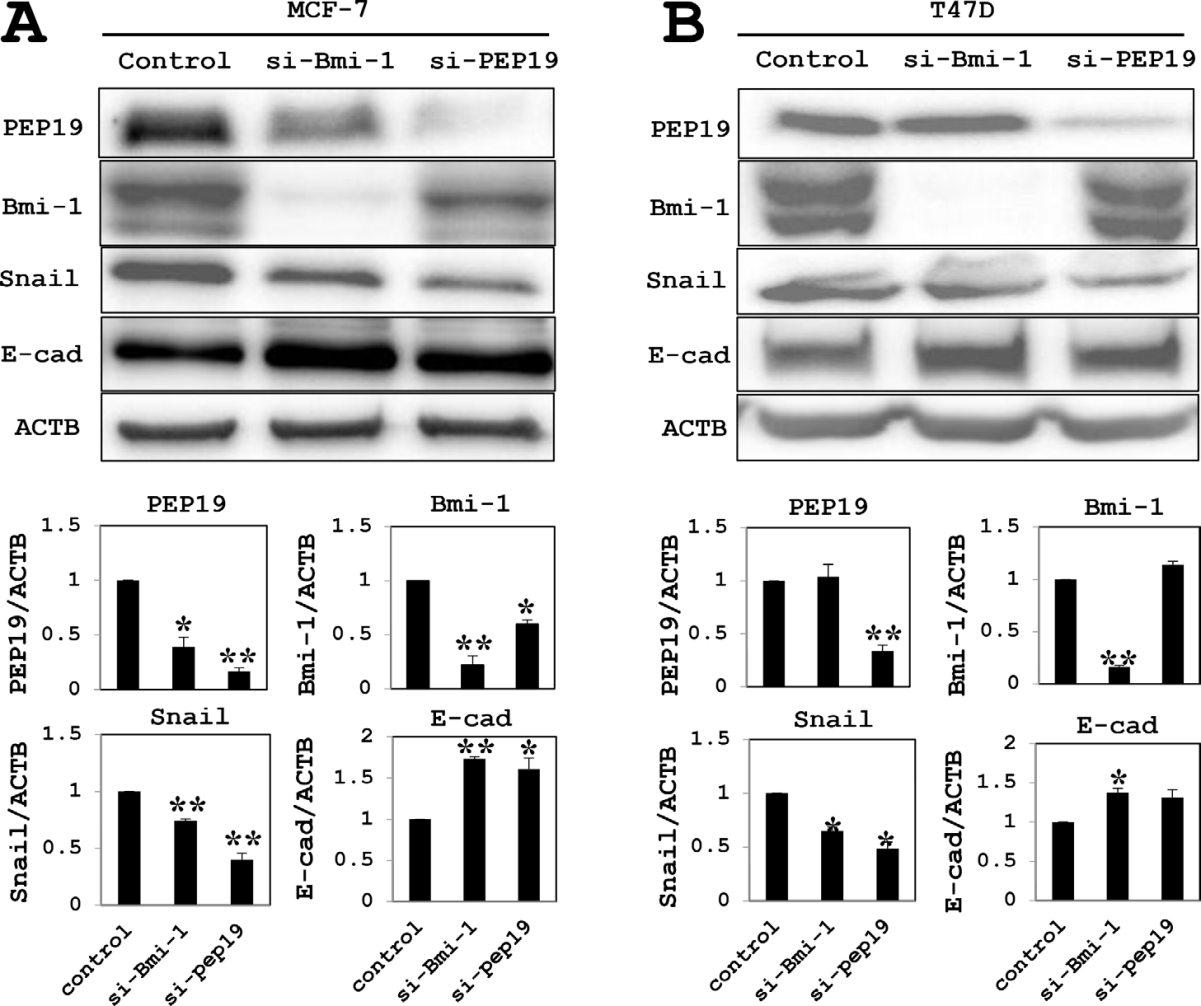
Western blot analysis of EMT marker expression (**A**) In MCF-7 cells (cultured in 10^−9^ M E2-containig medium), PCP4/PEP19 and Bmi-1 protein levels were reduced after knockdown of Bmi-1 and PCP4/PEP19, respectively. Snail and E-cadherin levels were decreased and increased, respectively, by Bmi-1 and PCP4/PEP19 knockdown. (**B**) Knockdown experiments of Bmi-1 and PCP4/PEP19 in T47D cells (cultured in 10^−8^ M E2-containig medium) showed similar results to those obtained in MCF-7 cells, except that the E-cadherin expression was not significantly increased after PCP4/PEP19 knockdown. The expression of each protein was normalized to that of β-actin (ACTB) (*n* = 4). *p < 0.05 and **p < 0.01 vs. control. E-cad, E-cadherin; si-Bmi-1, Bmi-1 knockdown; si-PEP19, PCP4/PEP19 knockdown.

**Figure 5:**
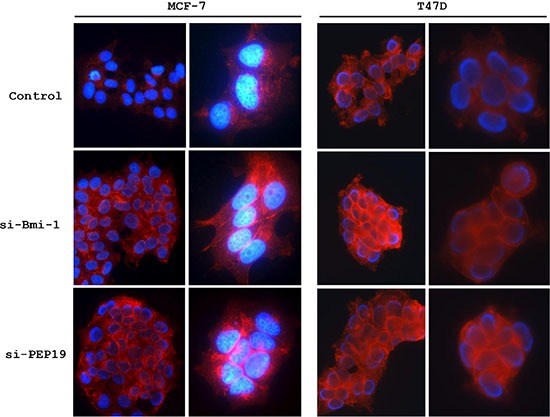
Immunocytochemical analysis of E-cadherin expression E-cadherin expression was upregulated in MCF-7 and T47D cells Bmi-1 or PCP4/PEP19 knockdown. si-Bmi-1, Bmi-1 knockdown; si-PEP19, PCP4/PEP19 knockdown.

In MCF-7 cells, loss of Bmi-1 reduced the mRNA and protein expression of PCP4/PEP19, whereas PCP4/PEP19 knockdown decreased Bmi-1 mRNA and protein expression (Figures 3C and 4A). Loss of Bmi-1 inhibited the expression of Snail and increased that of E-cadherin relative to control-transfected cells. A similar trend was observed upon PCP4/PEP19 knockdown (Figure 4A). An immunocytochemical analysis revealed an increase in membrane expression of E-cadherin in cells treated with Bmi-1 or PCP4/PEP19 siRNA (Figure 5). In T47D cells, PCP4/PEP19 and Bmi-1 knockdown did not change the expression of Bmi-1 and PCP4/PEP19, respectively (Figure 3D). Snail expression was decreased by Bmi-1 and PCP4/PEP19 knockdown (Figure 4B). E-cadherin expression was significantly increased by knockdown of Bmi-1, but not by that of PCP4/PEP19 (Figure 4B). The cellular distribution, however, appeared to be increased at the cell membrane after PCP4/PEP19 knockdown as well as Bmi-1 knockdown (Figure 5).

### Loss of Bmi-1 and PCP4/PEP19 suppresses cell migration and invasion

**Figure 6:**
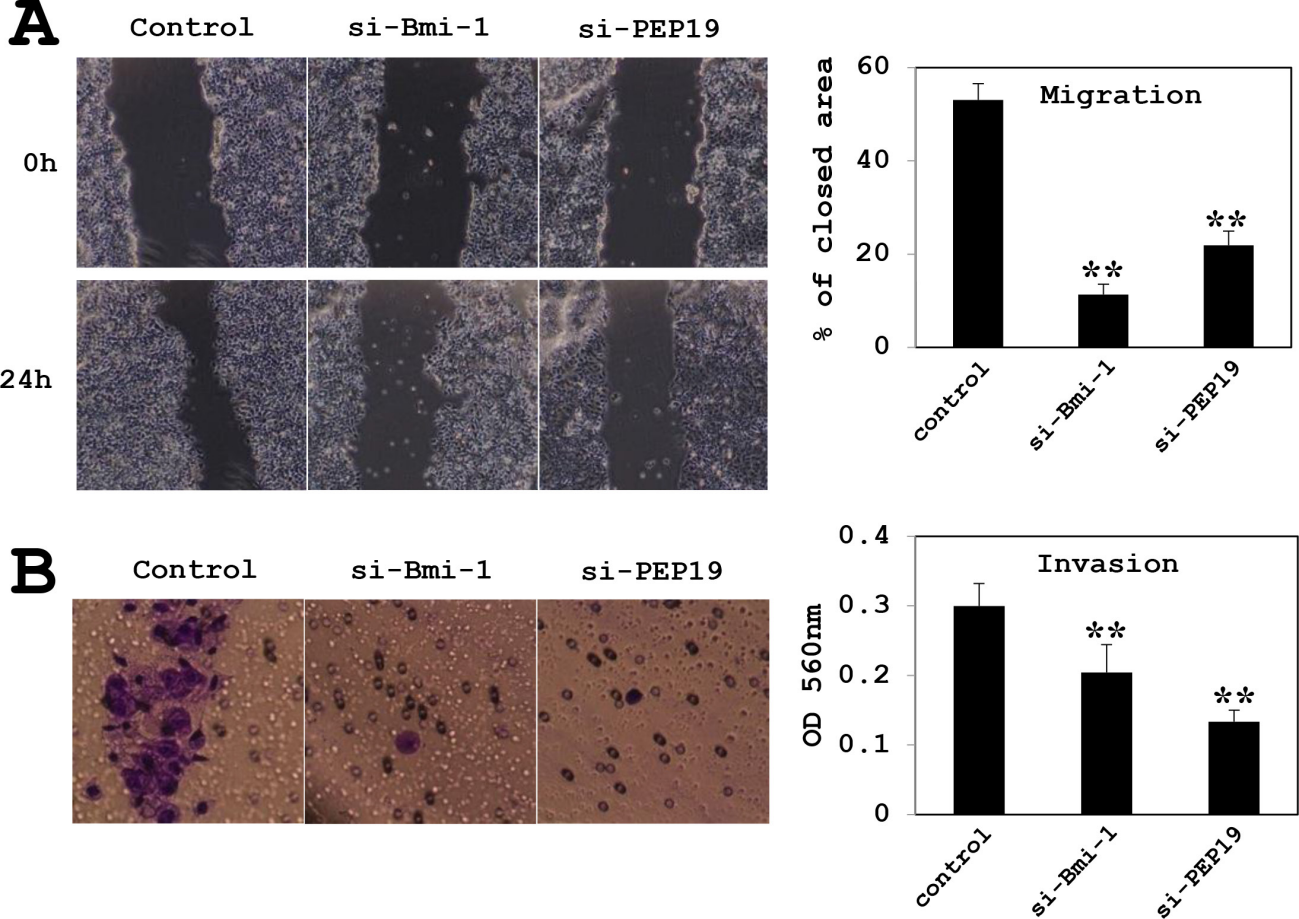
Effects of Bmi-1 and PCP4/PEP19 knockdown on cell migration and invasion in MCF-7 cells (**A**) Cell migration was monitored by the wound healing assay (*n* = 6). Areas covered by migrating cells 24 h after scratching the surface of the plate were measured; areas were decreased by Bmi-1 and PCP4/PEP19 knockdown relative to the control. (**B**) Invasion was measured using the Boyden chamber method (*n* = 8). After 24 h of culture in the chamber, cells that had penetrated the pores were counted. Invasion was markedly reduced by Bmi-1 and PCP4/PEP19 knockdown. **p < 0.01 vs. control. si-Bmi-1, Bmi-1 knockdown; si-PEP19, PCP4/PEP19 knockdown.

**Figure 7:**
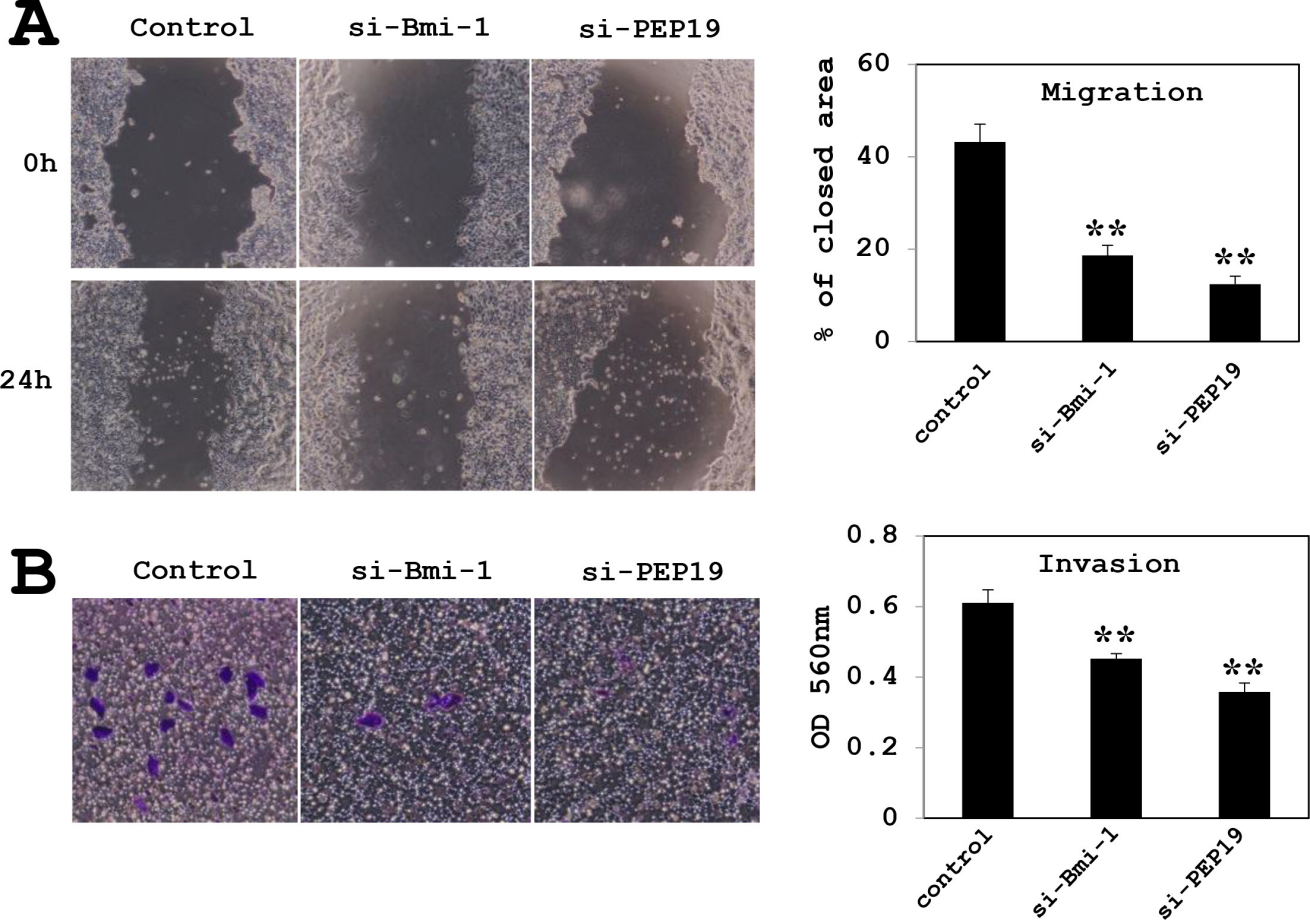
Effects of Bmi-1 and PCP4/PEP19 knockdown on cell migration and invasion in T47D cells (**A**) Cell migration was monitored by the wound healing assay (*n* = 6). (**B**) Invasion was measured using the Boyden chamber method (*n* = 8). **p < 0.01 vs. control. si-Bmi-1, Bmi-1 knockdown; si-PEP19, PCP4/PEP19 knockdown.

MCF-7 cell migration was assessed with the wound-healing and invasion assays. In cells treated with control siRNA, approximately 50% of the initial wound area was repaired by migrating cells after 24 h; however, in Bmi-1 and PCP4/PEP19 knockdown cells, only 10% of the area, was repaired (Figure 6A). A similar result was obtained with the invasion assay; that is, cell invasion through basement membrane-coated pores was decreased upon Bmi-1 and PCP4/PEP19 knockdown relative to control cells (Figure 6B). The experiments using T47D cells showed a similar results (Figure 7).

### Effects of PCP4/PEP19 and Bmi-1 knockdown on RhoA, Rac1 and Cdc42 activities

**Figure 8:**
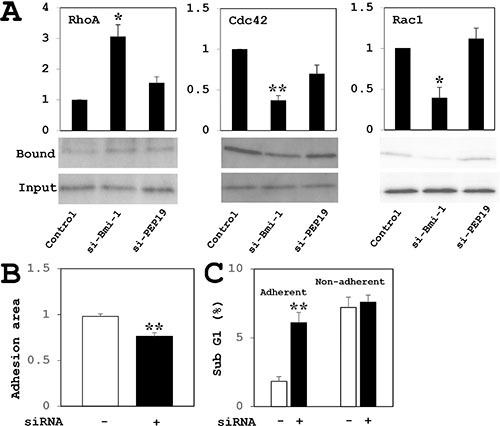
(**A**) Analysis of RhoA, Cdc42, and Rac1 GTPase activities in MCF-7 cells. RhoA activity was increased and Cdc42 and Rac1 activities were decreased by Bmi-1 knockdown relative to controls. PCP4/PEP19 knockdown had no effect on GTPase activity (*n* = 4). Cell were stimulated with cultured in 10^−8^ M E2 for 15 min after overnight starvation. (**B**–**C**) Effects of PCP4/PEP19 knockdown on cell adhesion in MCF-7 cells. Cells were trypsinized 72 h after siRNA transfection and allowed to attach onto new culture dishes. (B) Adherent cell areas were measured 8 h later by crystal violet staining (*n* = 6). (C) The percentage of apoptotic cells in the adherent fraction was increased upon PCP4/PEP19 knockdown as compared to control siRNA transfection. In the non-adherent fraction, there was no difference in the percentage of apoptotic cells between two groups (*n* = 3 to 5). *p < 0.05 and **p < 0.01 vs. control siRNA-transfected cells.

PCP4/PEP19 knockdown did not change the protein expression and activities of RhoA, Rac1 and Cdc42 GTPases in MCF-7 cells. In contrast, RhoA activity was increased and those of Rac1 and Cdc42 were decreased by Bmi-1 knockdown. (Figure 8A).

### Loss of PCP4/PEP19 perturbs cell adhesion

The MCF-7 cells transfected with negative control siRNA attached to the surface of culture dishes; however, some of PCP4/PEP19-silenced cells were non-adherent (Figure 8B). The sub-G1 fractions of adherent cells was increased by PCP4/PEP19 knockdown (Figure 8C, left two columns). In contrast, there was no difference in the percentage of cells in the sub-G1 fraction of non-adherent cells between negative control and PCP4/PEP19 siRNA-treated cells (Figure 8C, right two columns).

## DISCUSSION

PCP4/PEP19 has an anti-apoptotic function in human breast cancer cell lines [16]. In the present study, we demonstrated that loss of PCP4/PEP19 expression decreased cell adhesion, migration, and invasion in MCF-7 and T47D human breast cancer cells. These events preceded apoptosis and was accompanied by aggregation and alterations in cell morphology, including loss of filopodia-like structures and focal adhesion complexes as well as decreased vinculin expression. These results suggest that PCP4/PEP19 is required for activities of cancer cell migration, invasion, and adhesion.

EMT is a complex process by which epithelial cells acquire a mesenchymal phenotypes, which includes the loss of adhesion and increased motility [20, 21]. EMT plays a critical role in organ development, tissue remodeling, and in cancer invasion and metastasis [22–24]. Many studies have linked EMT to the invasive and metastatic potential of breast cancer cells [25, 26]. A feature of EMT is suppression of E-cadherin expression, which disrupts cell-cell adhesion and activates signaling pathways that control cell migration, invasion, and metastasis [27, 28]. Bmi-1 is an upstream regulator of Snail expression, which in turn promotes the EMT via suppression of E-cadherin and upregulation of vimentin expression [29, 30]. In the present study, E-cadherin levels were increased by knockdown of Bmi-1 and PCP4/PEP19, suggesting that the factors may enhance EMT by acting in the same pathway(s). Loss of Bmi-1 has been reported to inhibit cell proliferation and enhance apoptotic cell death, which decreases Akt phosphorylation in MCF-7 cell [31]. Taken together with our previous results demonstrating that phosphorylation of Akt is reduced by PCP4/PEP19 knockdown [16], our current findings implicate PCP4/PEP19 as a novel factor in the upregulation of EMT in human breast cancer. In addition, the expression of both PCP4/PEP19 and Snail was inhibited by Bmi-1 knockdown, suggesting that PCP4/PEP19 acts downstream of the Bmi-1 signaling pathway in MCF-7 cells but not in T47D cells.

Among Rho family small GTPases, RhoA, Rac1, and Cdc42 have been extensively studied for their roles in regulating cell motility and migration via actin reorganization and alteration of membrane structures [32]. Cdc42 and Rac1 GTPases stimulate the formation of filopodia and lamellipodia, respectively [33–35], while RhoA induces actin stress fiber formation and promote the maturation of adhesion complexes [36]. Although Rac1/Cdc42 and RhoA have antagonistic functions [37], their coordination of their activities is essential for cell motility and cancer metastasis [38]. In our study, RhoA activity was increased and those of Rac1 and Cdc42 were decreased by Bmi-1 knockdown in the MCF-7 cells. In contrast, loss of PCP4/PEP19 had no effect on the activity of these GTPases, despite the fact that PCP4/PEP19 knockdown suppressed cell migration and invasion. These results suggest that PCP4/PEP19 does not involve the regulation of these GTPases activities, even though PCP4/PEP19 may be a downstream signaling of Bmi-1.

In metastasis, cancer cells detach from the interstitial extracellular matrix and invade the stroma and vasculature, adhering to endothelial cells and stroma at remote sites and undergoing proliferation. Thus, unlike normal epithelial cells, cancer cells can survive even in without adhesion in the lymph and blood stream [39, 40]. Anoikis is the process by which apoptosis occurs as a result of loss of adhesion to adjacent cells or the extracellular matrix [40, 41]. Epithelial cells are more susceptible than fibroblasts to anoikis; indeed, normal MCF-10A mammary epithelial cells undergo anoikis following loss of cell attachment, whereas MCF-7 cells show resistance [42]. Therefore, the sensitivity of cells to anoikis is inversely associated with their capacity for transformation [41]. Knockdown of PCP4/PEP19 resulted in cell aggregation and decreased migration, invasion, and cell adhesion, but in no increased apoptosis in non-adherent cell fractions, indicating that PCP4/PEP19 may be irrelevant to anoikis.

In conclusion, we report a novel role for PCP4/PEP19 in the upregulation of cell motility in addition to its previously described anti-apoptotic function in human breast cancer cells. Given that increased motility and suppression of apoptosis promotes cancer cell survival, these results suggest that PCP4/PEP19 can potentially serve as a molecular therapeutic agent designed to suppress breast cancer cell proliferation, invasion, and metastasis.

## METHODS

### Cells and cell culture

MCF-7 and T47D human breast cancer cells were obtained from RIKEN BioResource Center (Tsukuba, Japan) and ATCC, respectively. The cells were grown in minimal essential medium (MEM, Sigma, St. Louis, MO) or RPMI1640 medium (Sigma) containing 10% FBS and maintained at 37°C in 95% air and 5% CO_2_. Steroid hormones were removed from FBS by incubation in a 5% charcoal and 0.5% dextran suspension at 45°C for 1 h. The suspension was centrifuged at 2,500 rpm, and the supernatant was passed through a 0.2 μM filter. In each experiment, cells were cultured with medium containing 10^−8^ M or 10^−9^ M 17-β estradiol (E2, Sigma) and 10% charcoal-stripped FBS and without phenol red.

### Antibodies and reagents

Anti-PCP4/PEP19 polyclonal rabbit antibody was purchased from Sigma. Antibodies against Snail, vinculin, and E-cadherin were from Abcam (Burlingame, CA). Rabbit monoclonal antibody against Bmi-1, β-actin (ACTB), histone deacetylase 1 (HDAC1) and Alexa Fluor 594- and 488-conjugated secondary antibodies were from Cell Signaling Technology (Danvers, MA). FITC-conjugated phalloidin (Acti-stain Fluorescent Phalloidin) and kits for active RhoA/Rac1/Cdc42 pull down assays were from Cytoskeleton Inc. (Denver, CO). Lipofectamine RNAiMAX was obtained from Life Technologies (Carlsbad, CA).

### SiRNA knockdown experiments

Pre-designed siRNAs were used to knock down PCP4/PEP19 (ID: HSS181928) and Bmi-1 (IDs: HSS101038, HSS101039, and HSS101040), and Stealth RNA siRNA (Life Technologies) was used as a negative control. Cells were transfected with siRNAs using Lipofectamine RNAiMAX in phenol red-free MEM (Life Technologies) containing 10% charcoal-stripped FBS. The following day, the medium was changed to phenol red-free MEM containing 10% charcoal-stripped FBS and E2.

### Western blot analysis

Cells were washed with cold PBS and precipitated with 10% trichloroacetic acid on ice for 30 min. Precipitates were washed with PBS and dissolved in lysis buffer composed of 50 mM Tris-HCl (pH 6.8), 2% SDS, 10% glycerol, 6% 2-mercaptoethanol, and 0.01% bromophenol blue. Lysates were resolved on Tris/Tricine or Tris/glycine gels and transferred to PVDF membranes, which were blocked with 5% milk in TBS (pH 7.6) with 0.1% Tween 20. The membranes were incubated overnight at 4°C with primary antibodies diluted in Can Get Signal solution 1 (Toyobo, Osaka, Japan), followed by horseradish peroxidase-conjugated goat anti-rabbit or anti-mouse antibody (MP Biomedicals, Santa Ana, CA). Protein expression was detected with SuperSignal West Pico chemiluminescent substrate or SuperSignal West Femto Maximum Sensitivity Substrate (Thermo Scientific, Waltham, MA). For subcellular fractionation of PCP4/PEP19 expression, the cell lysates were fractioned into cytoplasmic and nuclear fractions using a kit (NE-PER™ nuclear and cytoplasmic extraction reagents, Thermo Scientific) and applied to Western blotting. Densitometry analysis was performed using CS Analyzer 3.0 (ATTO, Tokyo, Japan).

### Quantitative real-time PCR analysis of gene expression

Total RNA was extracted using ReliaPrep RNA Cell Miniprep System (Promega, Maddison, WI) and was converted into cDNA using a High Capacity RNA-to-cDNA kit (Life Technologies). The cDNA was analyzed on a LightCycler 480 (Roche Diagnostics, Basel, Switzerland) using TaqMan assay (Life Technologies). Each sample was analyzed in triplicate in separate wells to determine target (PCP4/PEP19 and Bmi-1) and reference (18S rRNA) gene expression. The average of three threshold cycle values was calculated for each gene and analyzed with the comparative Ct method. Custom made primers and TaqMan probes were purchased from Life Technologies (assay IDs: Hs01113638_m1 and Hs00995536_m1, respectively).

### Wound healing assay

After siRNA knockdown, the cells were cultured in 10^−9^ M E2-containing medium until confluence (36 h) in 24-well plates and starved in serum free medium for overnight. Cell monolayer were stripped using a pipette tip, washed with PBS, and incubated with 10^−9^ M E2-containing fresh medium for 24 h. Wound areas were measured using Image J software.

### Invasion assay

Cell invasion was evaluated with the CytoSelect 24-Well Cell Invasion Assay kit (Cell Biolabs, San Diego, CA). After siRNA knockdown, the cells were cultured in 10^−9^ M E2-containing medium for 36 h and starved in serum free medium for overnight. Then, 3 × 10^5^ cells in serum-free medium were seeded on a basement membrane-coated insert with 8 μM pores, and 500 μl of medium containing 10% of charcoal-stripped FBS and E2 (10^−9^ M) were added to the lower chamber. After a 24-h incubation, cells that had penetrated the insert pore were extracted and absorbance at OD560nm was measured.

### Immunocytochemistry

Cells grown in 4-well Lab-Tek Chambered slides (Nunc/Nalgene, Penfield, NY) were stimulated with 10^−8^ M E2 for 15 min after overnight starvation, and fixed with 10% buffered formalin. After washing with PBS, cells were permeabilized by treatment with 0.5% Triton X-100. F-actin filaments were visualized by FITC-conjugated phalloidin staining. Vinculin expression demonstration was detected using a primary antibody and Alexa Fluor 594-conjugated secondary antibody and visualized by a conventional fluorescence microscopy (Olympus, Tokyo, Japan). For PCP4/PEP19 demonstration, the cells cultured on glass slides were fixed with ice cold acetone and incubated with anti-PCP4/PEP19 antibody. The cellular localization was visualized by Alexa Fluor 488-conjugated secondary antibody.

### Active RhoA, Rac1, and Cdc42 pull-down assay in MCF-7 cells

MCF-7 cells were stimulated by 10^−8^ M E2 for 15 min after Bmi-1 and PCP4/PEP19 knockdown and overnight starvation. Total lysates were incubated with GST-rhotekin-Rho-GTP-binding domain beads to pull down active RhoA and with GST-p21-activated protein kinase-p21-biding domain beads to pull down active Rac1/Cdc42 according to the manufacturer's instruction. Active proteins were detected by western blotting using the total cell lysates.

### Cell adhesion assay in MCF-7 cells

Cells maintained in the growth medium (10^−9^ M E2) were trypsinized 72 h after transfection of control or PCP4/PEP19 siRNA and re-plated in 24-well plates at 1 × 10^5^ cells/well; 8 h later, adherent cells were washed twice with PBS, fixed with methanol, and stained with 1% crystal violet. Adherent cell areas were measured using ImageJ software. Cell apoptosis of adherent and non-adherent cells was evaluated by flow cytometry, as previously described [16].

### Statistical analysis

Data are presented as mean ± SE. Statistical significance was determined with the unpaired one-tailed Student's *t* test. *p* < 0.05 was considered statistically significant.

## Abbreviations

Purkinje cell protein 4/peptide 19: PCP4/PEP19
epithelial-mesenchymal transition: EMT
